# The Molecular Mechanism of Polyphenols with Anti-Aging Activity in Aged Human Dermal Fibroblasts

**DOI:** 10.3390/molecules27144351

**Published:** 2022-07-07

**Authors:** Joo Hwa Lee, Jooho Park, Dong Wook Shin

**Affiliations:** 1 College of Biomedical and Health Science, Konkuk University, Chungju 27478, Korea; hesiit@kku.ac.kr; 2 Department of Applied Life Science, Graduate School, BK21 Program, Konkuk University, Chungju 27478, Korea; pkjhdn@kku.ac.kr

**Keywords:** dermal fibroblast, aging, molecular mechanism, polyphenol, ultraviolet

## Abstract

Skin is the largest organ in the body comprised of three different layers including the epidermis, dermis, and hypodermis. The dermis is mainly composed of dermal fibroblasts and extracellular matrix (ECM), such as collagen and elastin, which are strongly related to skin elasticity and firmness. Skin is continuously exposed to different kinds of environmental stimuli. For example, ultraviolet (UV) radiation, air pollutants, or smoking aggravates skin aging. These external stimuli accelerate the aging process by reactive oxygen species (ROS)-mediated signaling pathways and even cause aging-related diseases. Skin aging is characterized by elasticity loss, wrinkle formation, a reduced dermal-epidermal junction, and delayed wound healing. Thus, many studies have shown that natural polyphenol compounds can delay the aging process by regulating age-related signaling pathways in aged dermal fibroblasts. This review first highlights the relationship between aging and its related molecular mechanisms. Then, we discuss the function and underlying mechanism of various polyphenols for improving skin aging. This study may provide essential insights for developing functional cosmetics and future clinical applications.

## 1. Introduction

The skin dermis is composed of the upper papillary layer and the lower reticular layer. The papillary layer includes abundant fibroblasts, blood vessels, and phagocytes, while the reticular layer includes mainly collagen fibers in the dermal matrix [1]. The dermis is also comprised of blood vessels, nerve endings, and the immune system, such as mast cells and macrophages [1]. Skin is constantly exposed to various oxidative stress, and skin aging is an inevitable process. Skin aging can be classified into intrinsic aging and extrinsic aging [2,3,4]. Intrinsic aging is a consequence of physiological changes that occur naturally as we age. Especially, the ability of dermal fibroblasts to synthesize collagen attenuates with age and leads to a severe decline in the integrity of collagen fibers. Extrinsic aging is a consequence of continuous exposure to the external environment including ultraviolet (UV) irradiation and air pollution [2,3,4,5]. Especially, UV radiation is a major causative factor of inflammatory responses, DNA damage, and various cutaneous lesions such as skin photoaging. 

Many studies have reported various harmful effects of UV on the dermis [6,7]. The photoaged dermis is generally characterized by disorganized or fragmented collagen fibers, and the degradation of elastic fiber, which results in wrinkle formation, delayed wound healing, and sagging [6,7,8]. Damaged collagen fibrils and elastin fibers in the UV-mediated dermis are mainly caused by the matrix-degrading metalloproteinases (MMPs) synthesis. MMPs are a family of endopeptidases and take part in inflammatory processes by modulating chemokine activity [6,7,8]. In addition, air pollution such as particulate matter 2.5 (PM 2.5) also causes skin damage and results in oxidative stress, inflammation, and even premature skin aging [5,9]. 

For this reason, protection from extrinsic or intrinsic aging is an essential issue in the cosmeceutical and dermatological fields. Novel active ingredients are required to retard or prevent skin aging by suppressing the harmful effects of UV. Especially, many researchers have demonstrated that natural polyphenols worldwide can be identified as a potential active ingredient to improve the aged skin dermis [10,11,12]. Polyphenol is a type of aromatic alcohol compound found in plants and is characterized by having several hydroxyl groups with a functional group of two or more phenyl groups [13,14,15]. Polyphenols are the ingredients of pigments and cause the bitterness of plants produced by photosynthesis, so they are as clear as grapes and there are many in foods that are silvery or bitter. In addition to catechin in green tea, quercetin in apples and onions, and anthocyanin are also known. Polyphenols are classified into principal classes: “flavonoids, stilbenes, phenolic acids, and lignans”. Flavonoids account for the majority of polyphenols. Flavonoids include flavones, flavonols, flavanols, flavanones, isoflavones, and anthocyanins. Many studies demonstrated that polyphenols have the antioxidant effect of scavenging reactive oxygen species (ROS) and enhance the autophagy process for improving the aging process [13,14,15]. In this review, we investigated the recent anti-aging effects of polyphenols and their mechanisms and propose potential insights for improving aged dermal fibroblasts in the future.

## 2. Results 

### 2.1. Molecular Mechanism in Aged Dermal Fibroblasts 

#### 2.1.1. Crosstalk between Reactive Oxygen Species and Inflammation 

**Figure 1:**
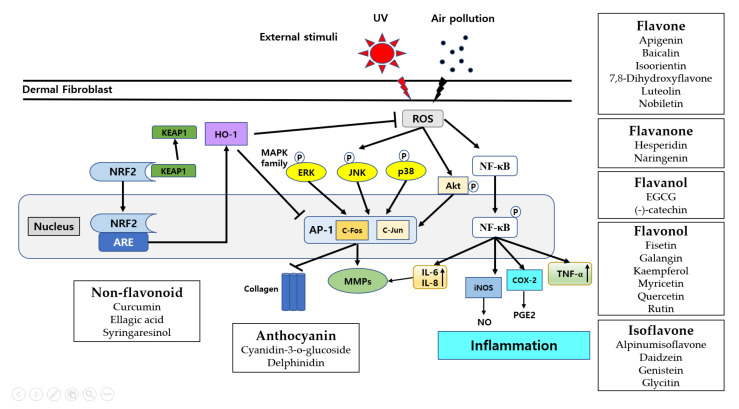
Diagram of several molecular mechanisms in skin dermis exposed to external stimuli. External stimuli such as UV radiation or air pollutants can cause direct damage to the DNA and produce ROS. These can further stimulate many inflammatory responses and the MAPK family, which can lead to photoaging through inflammation and collagen degradation. The KEAP1-NRF2 stress response pathway is the principal inducible defense against oxidative stresses. Under homeostatic conditions, KEAP1 regulates the activity of NRF2. In response to stress, an intricate molecular mechanism facilitated by sensor cysteines within KEAP1 allows NRF2 to escape ubiquitination, accumulate within the cell, and translocate to the nucleus, where it can promote its antioxidant transcription program. In the diagram, polyphenol chemicals in each box are organized into the classification system of polyphenol. UV, Ultraviolet; ROS, reactive oxygen species, HO-1; heme oxidase.

External stimuli such as UV irradiation or air pollutants can generate ROS, which causes an imbalance between ROS production and antioxidant mechanisms, leading to causing oxidative stress [3,6]. This oxidative stress is an important factor regulating dermal alteration in the aging process. This oxidative stress can also initiate pro-inflammatory cytokines such as tumor necrosis factor α (TNF-α) and interleukin-6 (IL-6), which play a key role in the inflammatory response [3]. NF-κB is a protein complex responsible for immune responses, and its dysregulation is involved in various diseases, such as inflammation and aging [16]. Activated NF-κB subunits are translocated into the nucleus and cause upregulation of pro-inflammatory cytokine expression [16]. Activation of NF-κB can also induce the expression levels of MMPs [6,16]. These external stimuli also increase the cellular levels of nitric oxide (NO) and prostaglandin E2 (PGE_2_) by activating iNOS and COX-2, respectively. The expression levels of pro-inflammatory cytokines including TNF-α and IL-6 are remarkably increased in UV-irradiated human dermal fibroblasts (HDFs) [3,6,16]. These external stimuli also lead to an increase in the phosphorylation of the MAPK family such as extracellular signal-regulated kinase (ERK), c-jun *N*-terminal kinase (JNK), and p38, which then affects the phosphorylation of nuclear transcription factor AP-1 (c-Jun, c-Fos), which contributes to express MMPs [3,6,8]. On the contrary, many cells including dermal fibroblasts have a system to defend ROS called the nuclear factor erythroid-2-related factor 2 (NRF2) signaling pathway [17]. This pathway accelerates the expression levels of genes that regulate processes such as protein stability, autophagy, senescence, and protection against oxidative stress and inflammation. NRF2 is present in the cytoplasm as an inactive complex bound to its repressor, Kelch-like ECH-associated protein 1 (KEAP1). The dissociation of NRF2 from KEAP1 occurred in response to a stressful insult. In response to UV irradiation, the antioxidant response by NRF2 activation promoted the expression of detoxifying enzymes such as heme oxygenase 1 (HO-1) and cellular antioxidants [18] (Figure 1). 

This mechanism has long been known in aged dermal fibroblasts, and the most common research on polyphenols has been used for products such as cosmetics and functional foods (Table 1). 

Several studies have demonstrated that several polyphenols ameliorated the harmful effects of UVA on aged dermal fibroblasts [2,19,20,21,22] (Figure 1). Apigenin, curcumin, cyanidin-3-o-glucoside (C3G), myricetin, and syringaresinol (SYR) decreased the expression of the MMP-1 in UVA-irradiated HDFs [2,19,20,21,22]. In an in vivo study, the apigenin-containing cream improved dermal density and skin elasticity and decreased fine wrinkle length [2]. Curcumin attenuated UVA-induced ER stress and inflammation signaling by reducing the protein expression of NF-κB [20]. C3G decreased the phosphorylation level of p38 but not JNK [19]. SYR suppressed the UV-induced phosphorylation of JNK and AP-1. In addition, SYR inhibited the UVA-induced secretion of IL-1β, IL-6, TNF-α, and COX-2 [22]. Myricetin inhibited the UVA-mediated activation of p38, ERK, and JNK [21]. Interestingly, myricetin balances the TIMP1/MMPs ratio and oxidative stress in diabetic fibroblasts, which causes foot ulceration in diabetic patients [23]. Quercetin remarkably stimulated NRF2 and enhanced the expression of HO-1 and catalase [24]. Interestingly, a ratio of 3:1 quercetin/curcuminoid mixture exhibited the maximal ability to activate the migration of fibroblasts [25]. 

Similarly, UVB-damaged fibroblasts were improved by several polyphenols [26,27,28,29,30,31,32] (Table 1). Baicalin, delphinidin, ellagic acid (EA), fisetin, isoorientin, genistein, and luteolin increased the expression levels of collagen I and III, whereas it decreased the expression levels of MMP-1 and MMP-3 [26,27,28,29,30,31,32]. Interestingly, baicalin had no difference in the normal fibroblasts without UVB irradiation [26]. Delphinidin significantly inhibited UVB-induced ROS generation and even nicotinamide adenine dinucleotide phosphate oxidase (NOX) activity by binding the NOX subunit [27]. EA recovered the total glutathione and superoxide dismutase activity levels and enhanced NRF2 activity [28]. Fisetin downregulated the phosphorylation levels of three MAPKs and inhibited the activation of NF-κB [30]. Isoorientin remarkably blocked JNK signaling activation [31]. Genistein strongly suppressed the production of IL-6 and MAPK signaling [33]. Hesperidin suppressed skin neovascularization by inhibiting the expression of vascular endothelial growth factor (VEGF), MMP-13, and MMP-9 in repetitive UVB-irradiated HR-1 hairless mice [34]. Luteolin diminished UV-induced ROS generation and the subsequent release of IL-6, IL-20 [35], COX-2, IL-1β, and TNF-α [36]. Luteolin also reduced UVB-induced erythema and wrinkle formation using the UVB irradiation of bare skin on the back of rats [37]. Rutin diminished UV-induced ROS generation and enhanced the activity/levels of SOD, plasma glutathione peroxidase (GSH-Px), and thioredoxin reductase (Trx) [38]. Interestingly, rutin significantly contributed to preventing the reduction in glutathione and vitamin E and C levels in UV-irradiated HDFs [38]. Rutin also ameliorated the alteration in the level of lipid mediators including malonaldehyde (MDA) and 4-hydroxynonenal (4-HNE) [38]. Rutin also partially prevented the UVA/B-induced increase in phosphatidylethanolamine and phosphatidyl-choline levels [39]. In addition, rutin restored phospholipase A2 activity and ROS generation, and the lipid peroxidation product, 4-hydroxynonenal level, increased UV-irradiated HDFs. Geogotek et al. demonstrated that the combination of ascorbic acid and rutin enhanced catalase and SOD. Interestingly, ascorbic acid stimulated UV-induced bilitranslocase activity necessary for transporting rutin, therefore accelerating the effect of rutin on the NRF2 pathway in UV-damaged fibroblasts [40]. 

Hydrogen peroxide (H_2_O_2_) is another harmful stimulus to accelerates the aging process in dermal fibroblasts [41,42,43,44,45] (Table 1). Galangin, genistein, kaempferol, and rutin recovered collagen I/III formation, and the expression of antioxidative proteins occurred in H_2_O_2_-damaged dermal fibroblasts [41,42,43,44,45]. Galangin remarkably reduced NF-κB activation, leading to a decrease in the expression of inflammatory factors, and modulating IGF1R/Akt-related proteins [41]. Genistein significantly improved the cell viability and mitochondrial membrane potential, while it increased glutathione (GSH) levels and the proliferation rate [43]. Rutin enhanced skin elasticity and downregulated the length, width, and many wrinkles in vivo [45].

In 12-O-tetradecanoylphorbol-13-acetate (TPA)-damaged dermal fibroblasts, kaempferol inhibited the phosphorylation of NF-κB, which is important for the IL-1β secretion and the expression of cleaved caspase-3 (Table 1). Kaempferol blocked the production of intracellular ROS and downregulated the phosphorylation level of JNK. Kaempferol also significantly inhibited bleomycin-induced oxidative stress in OKD48 mice [46]. Nobiletin inhibited the expression level of MMP-9 and suppressed the sustained activity of p38 in TPA-induced HDFs [47]. 

A tumor necrosis factor-α (TNF-α)-induced damaged fibroblast could also be alleviated by several polyphenols [48,49,50,51] (Table 1). Alpinumisoflavone (AIF), (-)-catechin, epigallocatechin-3-gallate (EGCG), and 7,8-dihydroxyflavone (7,8-DHF) suppressed the TNF-α-induced MMP-1 synthesis and enhanced procollagen I [48,49,50,51]. AIF and (-)-catechin inhibited NF-κB activity and COX-2 [48,49]. 7,8-DHF also significantly upregulated the expression of antioxidant enzymes including manganese superoxide dismutase (Mn-SOD), catalase, and heme oxygenase-1 (HO-1) [50]. EGCG also downregulated the phosphorylation level of ERK but not those of p38 and JNK [51]. Interestingly, EGCG has a beneficial effect against fine dust particle (FDP)-stimulated skin aging in HDFs [52]. 

#### 2.1.2. TGF-β/Smad Pathway 

**Figure 2:**
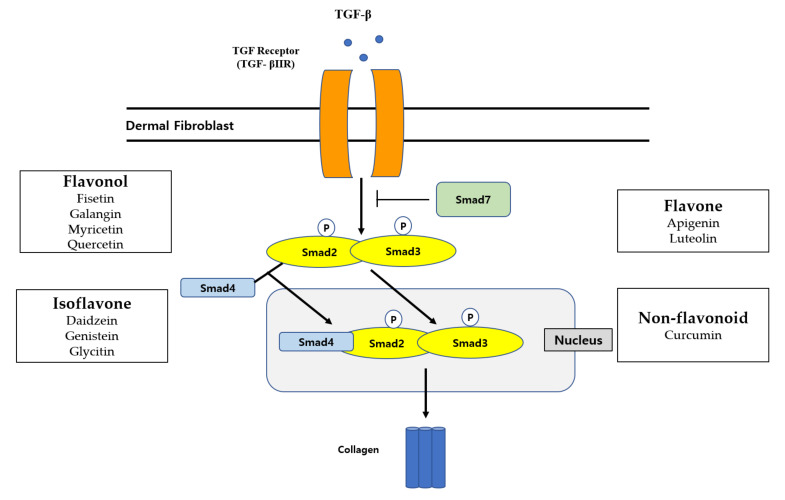
Diagram of TGFβ-mediated Smad2/3 signaling pathway and polyphenols in aged dermal fibroblasts.

Transforming growth factor-β (TGF-β) is a key regulator of ECM biosynthesis [53]. Especially, the TGFβ/Smad pathway is mainly responsible for the collagen synthesis in human dermal fibroblasts. TGF-β controls collagen homeostasis by regulating the Smad pathway [53]. First, TGF-β binds to a TGF-β type II receptor (TβRII), which can be associated with a TGF-β type I receptor (TβRI) and lead to its phosphorylation. This phosphorylation of TβRI induces the activation of both Smad2 and Smad3. Activated Smad2 or Smad3 bind to Smad4 for forming heteromeric Smad complexes. These Smad complexes move to the nucleus and interact with Smad-binding elements to induce the transcription process of procollagen genes [53,54]. UV irradiation can decrease procollagen synthesis by suppressing the TGF-β/Smad signaling pathway [55,56]. In addition to collagen synthesis, TGF-β/Smad signaling upregulates the expression level of ECM genes such as fibronectin, decorin, and versican, whereas it downregulates MMPs. This means that the TGF-β/Smad signaling pathway has an important role in maintaining the structural and mechanical integrity of dermal connective tissue by enhancing ECM production and inhibiting ECM degradation. Impaired TGF-β signaling leads to reduced collagen synthesis and causes a reduction in collagen levels (Figure 2). 

TGF-β binds to the TGF-β receptor, which enhances the phosphorylation level of Smad2/3. The Smad2/3 binds with Smad 4 and then moves to the nucleus. This pathway contributes to increasing collagen fibers. In the diagram, polyphenol chemicals in each box are organized into the classification system of polyphenols.

Various polyphenols have been studied to activate this TGF-β/Smad signaling pathway in aged dermal fibroblasts, which is important for the production of ECM (Table 1). Apigenin stimulated type-I and type-III collagen synthesis by activating the smad2/3 signaling pathway [57]. Glycitin also increased the phosphorylation levels of Smad2 and Smad3 [58]. Furthermore, glycitin also enhanced the phosphorylated form of AKT. Similarly, curcumin and daidzein also recovered UVA-damaged HDFs by increasing the protein expression of TGF-β and Smad2/3 [20,59]. Fisetin enhanced mRNA expression levels of CCN2 and Smad2, a CCN2 downstream mediator, dose-dependently [29]. In addition, fisetin treatment stimulated cell growth and proliferation in a time-dependent manner. Galangin ameliorated the H_2_O_2_/UVB-induced decrease in cell viability, the impairment of TGFβ/Smad signaling in H_2_O_2_/UVB-treated Hs68 cells, and dermal aging in UVB-induced C57BL/6J nude mice [60]. Interestingly, galangin suppressed the H_2_O_2_-induced expression of hsa-miR-4535, which is a candidate miRNA for targeting Smad4 and led to activating the Smad4 complex in HDFs. Topical application of galangin to the dorsal skin of C57BL/6J nude mice remarkably reduced UVB-induced skin photodamage by accelerating TGF-β/Smad collagen synthesis signaling, diminishing epidermal hyperplasia, and wrinkling. Interestingly, galangin also remarkably decreased the expressions of type I collagen, type III collagen, and TGF-β1, whereas it increased the expression of Smad7 in the HS rabbit ear model [61]. Genistein enhanced the thickness of collagen fibers by increasing TGF-β and tissue inhibitor of metalloproteinase (TIMP) expression levels [62]. Interestingly, luteolin selectively decreased the phosphorylation level of Smad2/3 in TGF-β/Smad signaling through binding to activin receptor-like kinase 5 (ALK5) and interfering with its catalytic activity [63]. 

#### 2.1.3. Senescence and Senolytic 

**Figure 3:**
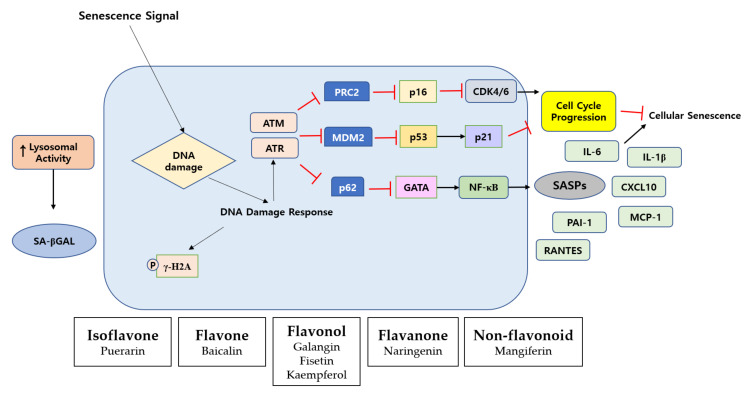
Diagram of senescence signaling pathway and polyphenols in aged dermal fibroblasts.

The main characteristics of senescent cells contain oxidative DNA damage, double-strand DNA breaks, and the impairment of DNA repair mechanisms. Compared to young cells, senescent cells exhibit a reduction in the extracellular matrix. The senescent cells exhibit increased cell-cycle inhibitors p21 and p16 and increased β-galactosidase activity, loss of nuclear high mobility group box 1 (HMGB1), and decreased lamin B1 [8,64]. These senescent cells produce senescence-associated secretory phenotypes (SASPs) such as pro-inflammatory cytokines and immune modulators [65]. Because these senescent cells have harmful effects on surrounding cells, recent strategies have aimed at the selective killing of senescent cells (called senolytic) or inhibiting SASPs without affecting the neighbor cells [66]. NF-κB has been considered to be a key factor in generating these SASPs [67,68] (Figure 3). 

UV irradiation is one of the key stimuli causing fibroblast senescence in vitro and in vivo [8,69]. Chronic UV radiation can cause a DNA damage response that can trigger cell cycle arrest through the p53/p21 pathway, and a significantly high accumulation of senescent cells [70,71]. This phenomenon can aggravate skin aging by secreting SASPs such as IL-6 and IL-8. These factors are responsible for chronic inflammation as well as ECM degradation [70,71]. 

Polyphenols such as flavonoids may prevent dermal fibroblasts from the aging process by targeting cellular pathways important for modulating cellular senescence and the secretion levels of SASPs (Table 1). Apigenin restored the viability of UVA-damaged HDFs and protects against the UVA-induced senescence of HDFs using a senescence-associated (SA)-β-gal assay [2]. Baicalin could also reduce the ratio of β-galactosidase-positive cells and p16, p21, and p53 expression in UVB-irradiated fibroblasts [26]. Interestingly, long-term baicalin incubation of UVB-induced senescent fibroblasts had no effects on cell proliferation. Galangin recovered H_2_O_2_/UVB-induced cell viability loss in HDFs [72]. The knockdown of SIRT1, PGC-1α, or NRF2 siRNA reversed the anti-aging effects of galangin. Furthermore, galangin diminished UVB-induced epidermal hyperplasia and activated the SIRT1/PGC-1α/NRF2 signaling pathway in the dorsal skin cells of C57BL/6J nude mice. Galangin could reverse the expression level of aging markers such as p53, p21^Cip1/WAF1^, p16^INK4A^, and senescence-associated β-galactosidase in H_2_O_2_-damaged Hs68 cells.

Senescence signals trigger DNA damage. Senescent cells are characterized by a DNA damage response, including chronic Ataxia Telangiectasia-mutated (ATM) and Ataxia Telangiectasia and Rad3-related (ATR) kinase signaling, which ultimately induces cell cycle arrest and senescence by activation of the p53/p21 and p16 pathways. In the diagram, polyphenol chemicals in each box are organized into the classification system of polyphenols.

Fisetin exhibited potent senolytic properties in vitro and in vivo. Administration of fisetin to old wild-type mice decreased the expression levels of p16 and p21, down-regulated the SASPs, and recovered tissue homeostasis by suppressing the PI3K/AKT/mTOR [73]. Kaempferol suppressed the induction of various SASP mRNAs in bleomycin-induced senescent fibroblasts and aged rats [74]. Mangiferin lowered the elevated ROS, stabilized the mitochondrial membrane potential, and downregulated the expression level of SA-β-gal in senescent HDFs [75]. Naringenin protected hairless mice from UVB-damaged skin by suppressing the secretion of SASPs such as IL-1β, IL-6, IL-10, TNF-α, and lipid hydroperoxides [76]. Puerarin enhanced cell proliferation and diminished the number of senescence-associated β-positive cells in senescent HDFs [77]. Puerarin downregulated the number of smooth muscle actin (SMA)-positive myofibroblasts and the expression of a reticular fibroblast marker, calponin 1 (CNN1), which were upregulated in senescent HDFs [77]. Recently, the combination of quercetin and dasatinib has been reported to remove senescent cells in vitro, improve physical function, and enhance the lifespan of mice in vivo [78]. 

#### 2.1.4. Autophagy

Autophagy is one of the conserved cellular processes that degrades damaged organelles or abnormal macromolecules to maintain cell survival and adaptation during starvation and oxidative stress [79]. Autophagy-related proteins Atg5, Atg12, and Atg16 and the 200 kDa family-interacting protein (FIP200), which make the mammalian complex after association with ULK1 and Atg13, are involved in the early phases of autophagy [80]. Then, ubiquitin-like Atg12 forms a complex with Atg5 by enzymatic conjugation to Atg7 and Atg10. The Atg5-Atg12 protein complex forms with Atg16. The complex then attaches to phagophores and detaches from mature autophagosomes. LC3 links to lipid phosphatidylethanolamine (PE) and is stimulated by Atg7 and Atg3 to generate LC3-II [80]. This LC3-II accelerates the targeted degradation of abnormal proteins and damaged cellular organelles by binding with adaptor proteins. A selective adaptor, p62, is attached to cargo proteins for the final degradation, and a targeted substrate is attached to LC3-II and the autophagosome and is used as a measurement index of autophagic flux [81]. Finally, LC3-positive autophagosomes are fused with lysosomes and lead to the degrading of a targeted substrate by lysosomal proteases [81,82]. Autophagy components are recycled in the cytosol and contribute to restoring important cellular processes after exposure to various stress factors and starvation. mTOR (mechanistic target of rapamycin), as a negative regulator of autophagy, integrates various signals and stress to regulate cellular metabolism. In contrast, 5’ adenosine monophosphate-activated protein kinase (AMPK), which is activated through an increase in the AMP/ATP ratio, stimulates the autophagy process [83]. 

Autophagy function and activity are reduced in aged human dermal fibroblasts because of the impaired degradation of autophagy [84]. Tashiro et al. demonstrated that impaired autophagic flux mainly caused the increased number of autophagosomes, which induced significant alteration in the composites of extracellular matrix proteins [84]. Repetitively UVA-irradiated HDFs downregulated autophagy through lysosome dysfunction [85]. The activation of autophagy aims to increase the degradation of metabolite adducts by UV irradiation-induced ROS and eventually leads to the inhibition of photoaging. 

Thus, several polyphenols have been reported to protect against photoaging by activation of autophagy (Table 1). Cyanidin-3-o-glucoside (C3G) can remarkably inhibit UVA-induced oxidative damage and apoptosis of HDFs [86]. The expression levels of Atg5 and LC3-II were remarkably diminished under 12 J/cm^2^. C3G recovered the levels of Atg5 and LC3-II in UVA-induced HDFs. To confirm this phenomenon, HDFs were pretreated with C3G and then treated with the autophagy inhibitor, 3-methyladenine (3-MA), after UVA irradiation of 12 J/cm^2^. 3-MA significantly decreased the inhibitory effects of C3G on morphological changes, oxidative damage, and apoptosis in UVA-damaged HDFs. The topical application of isoorientin ameliorated the UVB-damaged skin of mice by activating autophagy [31].

#### 2.1.5. DNA Damage and Repair 

DNA damage has been considered to be the primary cause of aging for a long time [87]. Many studies have demonstrated that the accumulation of DNA damage is involved with aging [88,89]. There are oxidative alterations, single- and double-strand breaks (DSBs), and various mutations in DNA damage [90]. DNA repair systems including base excision repair (BER), nucleotide excision repair (NER), mismatch repair (MMR), and double-strand break repair (DSBR) contribute to the repair of DNA damage. Unrepaired DNA damage during aging can cause genome instability and trigger a signal cascade that leads to cellular death or cellular senescence, and aging-related phenotypes [91]. In general, UVB irradiation generated the cyclobutane pyrimidine dimers (CPDs) in HDFs [92]. UVB-induced CPDs suppressed the expression levels of nucleotide excision repair (NER) genes including xeroderma pigmentosum complementation group proteins (XPC, XPB, XPG, and XPF) in HDFs. The capacity to repair DNA damage is reduced with aging [93]. There are a few studies regarding the effects of polyphenols on the DNA repair system in aged dermal fibroblasts (Table 1).

**Table 1:** Role of each polyphenol compound and its underlying mechanism for improving aged dermal fibroblasts.

Chemical Name	Group	Cell or Animal Type	Stimulus (Intensity)	Working Conc. (Max)	Mode of Action	References
Alpinum- isoflavone	Isoflavone	HDFs	TNF-α (20 ng/mL)	25–50 μM	↓ NF-κB, NOS ↓ COX2, AP 1	[48]
Apigenin	Flavone	HDFs, Women (>30 y)	UVA (25 J/cm^2^)	5–20 μM	↓ MMP-1 ↓ β-gal	[2]
		HDFs, NIH/3T3 C57B/6 mice	None	0.1–10 μM	↑ collagen I/III, ↑ smad2/3	[57]
		HDFs	UVB (20 mJ/cm^2^)	15 μM	↓ CPDs, ↓ XPB/C/G/, TFIIH	[92]
		HDFs BJ cells/ SD rat	UVA (25 J/cm^2^) Bleomycin (50 µg/mL)	5–10 μM 10, 20 μM	↓ NF-κB ↓ β-gal, SASPs	[74]
Baicalin	Flavone	C57BL/6 mice	UVB (0–240 mJ/cm^2^)	0.5, 1 mg/cm^2^ skin area	↑ collagen I/III ↓ MMP-1/3 ↓ β-gal, p53, ↓ p16, p21	[26]
Curcumin	Phenolic compound	HDFs	UVA (0–15 J/cm^2^)	0–10 μM	↓ ROS, MMP-1/3 ↓ NF-κB	[20]
Cyanidin-3-o-glucoside (C3G)	Anthocyanin	HDFs	UVA (0–12 J/cm^2^)	0–80 μM	↓ ROS, p38 ↑ Atg5, LC3II	[19,86]
(-) catechin	Flavanol	HDFs	TNF-α (20 ng/mL)	50, 100 μM	↓ MMP-1, ROS, ↓ MAPKs ↓ COX-2, IL-1β/-6	[49]
Daidzein	Isoflavone	HDFs BALB/C mice	None	0.5–50 μg/mL 200 μg/mL	↑ TGFβ/Smad, ↑ collagen I ↓ MMP-1	[59]
7,8 Di- hydroxyflavone	Flavone	Hs68	TNF-α (20 ng/mL)	0–10 μM	↓ ROS, MAPKs, Akt ↑ Mn-SODs, HO-1	[50]
Delphinidin	Anthocyanin	HDFs	UVB (20 mJ/cm^2^)	0–20 μM	↓ p38, JNK, ERK ↓ NOX	[27]
Ellagic Acid (EA)	Phenolic Lactone	HDFs	UVB (70 mJ/cm^2^)	0–30 μM	↓ MMP-2 ↑ Nrf-2	[28]
Epigallocatechin-3-gallate (EGCG)	Flavanol	Hs68	TNF-α (20 ng/mL)	10, 20 μM	↓ MMP-1, ERK	[51]
		HDFs	ERM-CZ100 (200 mg/mL)	12.5–50 μM	↓ ROS, MMPs, ↓ NF-κB, AP-1, ↓ MAPKs	[52]
Fisetin	Flavonol	HDFs	None	10–25 μM	↑ Smad2, CCN2, ↑ TGF-β1, β2, β3	[29]
		HDFs	UVB (40 mJ/cm^2^)	5–25 μM	↓ ROS, MMP-1,3,9 ↓ ERK, JNK, p38, ↓ NF-κB, COX-2, ↓ NO	[30]
		Murine DFs, HDFs C57BL/6 *p16*^Luc^	None	1–15 μM 500 mg/kg	↓ SA-β-gal ↓ SASPs	[73]
Galangin	Flavonol	Hs68	H_2_O_2_ (200 μM)	10–40 μM	↓ NF-κB, IL-6 ↑ collagen I/III	[41]
		HDFs/Hs68 C57BL/6J mice	H_2_O_2_ (20–40 M) UVB (150 mJ/cm^2^)	10, 30 µM 12,24 mg/kg	↑ NRF2, ↑ TGFβ/Smad ↑ SIRT1/PGC-1α ↓ p53, p16, p21	[60,61,72]
		New Zealand white rabbits ear HS Model	None	0.5–2 mg/mL	↑ TGF-*β*1, Smad 7 ↑ collagen I/III	[61]
Genistein	Isoflavone	HDFs	H_2_O_2_ (200 mM)	10, 100 μM	↑ GSH ↓ MAPKs ↓ NO, ROS	[43]
		HDFs Hairless male mice	UVB (100 mJ/cm^2^) UVB (200 mJ/cm^2^)	10 μM	↓ IL-6, MAPKs ↓ iNOS, COX-2	[31]
		OVX SD rats	None	1, 10 mg/kg (12 weeks)	↓ TGF-β1, VEGF, ↓ MMP-2, MMP-9	[62]
Glycitin	Isoflavone	HDFs	None	20 μM	↑ collagen I/III ↑ TGF-*β*1 ↓ MMP-1	[58]
Hesperidin	Flavanone	HR-1 hairless mice	UVB (20 mJ/cm^2^)	20 μM	↓ VEGF ↓ MMP-9/13	[34]
Isoorientin	Flavone	HDFs C57BL/6 mice	UVB (100 mJ/cm^2^)	40 μM	↓ MMP1, MMP3, ↓ JNK ↑ LC3II	[31]
Kaempferol	Flavonol	HDFs	TPA (5 µM)	100 nM	↓ IL-1β, ROS, JNK ↓ NF-κB, IκBα	[46]
		SSc fibroblast C57BL/6, OKD48 mice	H_2_O_2_ (0.5 mM) Bleomycin (300 µL/ug)	1, 10, 30 nM 40 mg/kg	↓ αSMA^+^, CD68^+^ ↓ HO-1, NOX2, ↓ IL-6, TNFα, ROS	[44]
		BJ cells SD rats	Bleomycin (50 µg/mL)	10, 20 μM	↓ NF-κB ↓ SA-β-gal, SASPs	[74]
Luteolin	Flavone	HDFs SD rats	UVB (300 mJ/cm^2^)	10, 20 μM	↓ ROS, MMPs, ↓ MAPKs ↑ collagen I	[32]
		Hs68	UVB (20 mJ/cm^2^)	20 μg/mL	↓ MMP-1, COX-2, ↓ IL-1β ↓ MAPKs, AP-1	[36]
		HDFs mouse HS	None	1–50 μM	↑ Smad2/3	[63]
		HDFs	CM from 6 J/cm^2^-HaCaT	8 ug/mL	↓ IL-20, IL-6 ↓ MMP-1, p38	[35]
Mangiferin	Xanthonoid	HDFs	H_2_O_2_ (10 µM) (15 days)	10, 50 μM	↓ SASPs ↑ ΔΨm	[75]
Myricetin	Flavonol	diabetic fibroblasts from the patient	None	3 μM	↑ TIMP1 ↑ catalase, SOD ↑ collagen I/III	[23]
		HDFs	UVA (10 J/cm^2^)	25 μM	↓ MMP-1, p38, ↓ ERK, JNK ↑ TGFβ/Smad	[21]
Nobiletin	Flavone	HDFs	TPA (200 nM)	5–50 μM	↓ MMP-9, p38	[47]
Naringenin	Flavanone	HDFs	UVA (6.3 J/cm^2^)	0.1, 0.05, 0.025%	↓ MMP-1 ↓ SA-β-gal ↓ SASPs	[76]
Rutin	Flavonol	HDFs from aged 30–50 years	H_2_O_2_ (0.2 mM)	100 µM	↓ ROS, MMP-1 ↑ collagen I	[45]
		CCD 1112Sk	UVA (20 J/cm^2^) UVB (200 mJ/cm^2^)	25 µM	↑ NRF2, catalase, ↑ SOD ↓ NF-κB	[38]
		CCD 1112Sk	UVA (20 J/cm^2^) UVB (200 mJ/cm^2^)	25 µM	↑ PE, PC ↑ linoleic acids, PLA2 ↓ ROS	[39]
		CCD 1112Sk	UVA (20 J/cm^2^) UVB (200 mJ/cm^2^)	25 µM	↓ ROS, MDA, ↓ 4-HNE, SOD ↑ GSH-Px, Trx ↑ vitamin E, GSH	[38]
Puerarin	Isoflavone	HDFs	25–35 Passages	25, 50 μM	↓ SA-β-gal ↓ SASPs	[77]
Quercetin	Flavonol	HDFs	UVA (10 J/cm^2^)	12.5 μM	↓ ROS ↑ HO-1, NRF2	[24]
		HDFs	None	Quercetin (5–25 μg/mL)/ Curcumin [(3:1)	↑ HDFs migration	[25]
Silibinin	Flavono-lignan	HDFs	UVB (1 mJ/cm^2^)	100 μM	↓ CPDs, XPA/B/C ↑ p53	[94]
Syringaresinol	Lignan	HDFs	UVA (10 J/cm^2^)	1, 5, 20 μM	↓ TNF-α, COX-2, ↓ IL-1β, IL-6 ↓ AP-1, MMP-1	[32]

Human dermal fibroblasts (HDFs), Conditioned medium (CM), membrane potential (ΔΨm), nicotinamide adenine dinucleotide phosphate (NAPDH) oxidase (NOX), Ovariectomized (OVX), Sprague-Dawley (SD) Rat, Phosphatidylethanolamine (PE), Phosphatidylcholine (PC), Phospholipase A2 (PLA2), Systemic sclerosis (SSc), Superoxide dismutase (SOD), fibroblast from mouse embryo (3T3-L1), 12-O-tetradecanoylphorbol-13-acetate (TPA), human foreskin fibroblast (BJ cells), “↑” increased; “↓” decreased.

Apigenin improved this UVB-induced loss of NER proteins in HDFs, meaning its protective effect against CPDs formation [92]. Interestingly, apigenin treatment prevented nuclear fragmentation, and apoptotic proteins, Bax and Caspase-3, in single low-dose UVB-irradiated HDFs. Apigenin also possessed a strong UV absorbance property and exhibited a 10.08 value of sun protection factor. Silibinin accelerated DNA repair by activating the NER pathway-related proteins such as XPA, XPB, XPC, and XPG in UVB-damaged HDFs [94]. Silibinin also increased the expression levels of p53 and GADD45α, which are the key factors of the NER pathway and DNA repair. Interestingly, silibinin exhibited no effect on UVB-irradiated DNA damage repair in XPA- and XPB-deficient HDFs, implying its important role in silibinin-mediated DNA damage repair. Furthermore, the DNA repair efficacy of silibinin was abolished in the presence of pifithrin-α, an inhibitor of p53. These data suggested that the efficacy of silibinin against UVB-induced photodamage is mainly processed by inhibiting NER and p53. 

## 3. Discussion 

Most scientific studies have focused on identifying natural polyphenols with various beneficial effects such as blocking ultraviolet rays, removing harmful oxygen, collagen synthesis, and preventing skin wrinkles. In this review, we provided information on several key molecular mechanisms in aged dermal fibroblasts and discussed natural polyphenols including many flavonoids, which have anti-aging effects and their molecular mechanisms. As described previously, the evaluation of polyphenol efficacy for aged dermal fibroblast has mainly focused on ROS, inflammation, and the TGF-β/Smad pathway. Recently, many studies on the effects of polyphenols on molecular mechanisms such as autophagy [15,95,96,97] and senescence [67,98,99,100] have been actively performed in various tissues. On the other hand, there are few studies on the effects of polyphenols on autophagy, senescence, and the DNA repair system in aged dermal fibroblasts. 

Most polyphenol compounds are usually stable and bioactive in plants. However, after the extraction from plants, these polyphenols are generally degraded because they are very sensitive to light or heat [101,102]. These polyphenols are also characterized by low solubility, bioavailability, and rapid metabolism. To increase their bioavailability and solubility, encapsulation technology such as liposomes is considered an efficient way to encapsulate polyphenol. This encapsulation retards the rapid degradation and regulates the optimal release of these polyphenols [101,102].

Thus, the research and development of these natural polyphenols in aged dermal fibroblasts should proceed as follows. First, the studies of polyphenols on autophagy, senescence, and the DNA repair system in aged dermal fibroblasts should be further progressed. Second, new aging biomarkers should be identified to understand dermal aging. Third, adequate formulations for the topical application of these effective natural polyphenols should be investigated and optimized regarding skin delivery improvement such as novel liposome technology. Fourth, clinical trials to maximize anti-aging efficacy by a combination of effective polyphenols or alone should be carried out. These studies may contribute to reducing oxidative stress, inflammation, and cellular damage in the aged dermis and can be used as an effective agent of cosmeceuticals for improving skin health. 

## 4. Materials and Methods

### 4.1. Search Strategy

Until 4 April 2022, we searched PubMed for published articles that investigated the effects of polyphenols on aged dermal fibroblasts. To reflect the latest research, the search timeframe was limited from 2012 year to the present (within 10 years). The search combined the keywords “polyphenol”, “flavonoid”, “lignan”, “tannic acid“, “aging”, “autophagy”, “senescence”, and “dermal fibroblast”. We also contained “liposome technology for the application of cosmetics”. 

### 4.2. Selection of Studies

Records were chosen by title and/or abstract to exclude studies that did not help answer the question in this review. Inclusion criteria: (1) published in English; (2) intervention included a flavonoid or polyphenol; (3) TGFβ/Smad, autophagy, senescence, or senolytic, or DNA repair.

### 4.3. Data Extraction

Data were extracted from selected studies (Table 1) as follows: (1) polyphenol source; (2) cell or animal type; (3) stimulus (or intensity); (4) polyphenol working concentration; (5) mode of action (or major molecular mechanism); (6) references.

## Data Availability

Not applicable.
